# Proregenerative Properties of ECM Molecules

**DOI:** 10.1155/2013/981695

**Published:** 2013-09-09

**Authors:** Stefan Plantman

**Affiliations:** Department of Neuroscience, Karolinska Institutet, 17177 Stockholm, Sweden

## Abstract

After traumatic injuries to the nervous system, regrowing axons encounter a complex microenvironment where mechanisms that promote regeneration compete with inhibitory processes. Sprouting and axonal regrowth are key components of functional recovery but are often counteracted by inhibitory molecules. This review covers extracellular matrix molecules that support neuron axonal outgrowth.

## 1. Introduction

The extracellular matrix (ECM) has a profound influences on individual cells and influences and/or controls several basic cellular processes such as adhesion, differentiation, survival, growth, and migration. In order to mount an effective regenerative response after axonal injury, the injured neuron must be able to initiate a number of changes at the cellular level including an increase in expression of relevant genes and protein transport with the aim of forming an extruding process known as a growth cone [1–3]. For functional recovery to occur, the growth cone must then successfully navigate back to its target by reacting to inhibitory and permissive cues in its surrounding and finally reestablishing proper connection with its original target. The ECM heavily influences all these processes.

When a peripheral nerve is injured, a series of cellular events collectively referred to as Wallerian degeneration occur. Macrophages invade the site of injury and in a coordinated effort together with Schwann cells start to clear the injured area of debris. A new neuronal growth cone is subsequently formed and starts to advance to its original target. The denervated Schwann cells proliferate and differentiate to a phenotype that aids in regeneration, by producing neurotrophic molecules, basement membrane components, and cell adhesion receptors. Schwann cells subsequently align along tubes of remaining endoneural basal lamina forming that so-called bands of Büngner. The growth cone advances in close contact with Schwann cells and the basal lamina. Although peripheral nervous system (PNS) lesions generally heal better than central nervous system (CNS) lesions, PNS lesions still cause significant physical impairment. It has been argued that current microsurgical techniques have reached a plateau where further advancements are unlikely to occur [4]. The situation is particularly complicated when a large portion of the nerve is lesioned and a gap occurs that requires nerve grafts harvested from another site (usually the sural nerve is used) that requires multiple surgeries and loss of function at another site. With this in mind, PNS regeneration is still a great clinical challenge, and current knowledge of factors that contribute to axonal regeneration could be of importance for tissue engineering strategies to develop artificial nerve grafts [5].

After a CNS lesion, retrograde neuronal cell death is generally more pronounced, and Wallerian degeneration is slower and less complete, although the inflammatory response is pronounced. In the CNS there are also other factors that contribute to the regenerative failure: (I) formation of fluid filled cysts, (II) lack of an organized basal lamina like the one seen in the PNS, and (III) the glial scar that is formed after a lesion inhibits growth cone advancement [6]. This scar is usually filled with inhibitory ECM molecules such as chondroitin sulphate proteoglycans. The current review has its focus on proregenerative matrix molecules, and for readers interesting in inhibitory ECM molecules, several excellent reviews are available [6–8]. 

## 2. Experimental Techniques to Study Neuronal Regeneration

### 2.1. Cell Cultures

The majority of the physiological findings on neuronal regeneration/outgrowth cited in the current study have been described using either cell-culture techniques or *in vivo* models of injury. Given the inherent vulnerability of adult CNS neurons to hypoxia, physical trauma, and dependence on cell-cell interaction, the majority of cell-culture studies cited in this review have relied on the culture of embryonic or early postnatal neurons from rodents, chick, or human. Since regenerative capacity of neurons diminishes with age [9, 10], caution must be taken when extrapolating these studies to the adult situation. The biggest exception to this being adult sensory neurons that are routinely cultured from adult animals in serum—and growth factor—free medium [11]. However, a recently developed protocol [12] has made it possible to successfully culture large numbers of neurons from the adult brain. This was achieved through a strategy whereby the dissociation and purification processes as well as the composition and pH of the culture media were optimized to improve survival and reach a high degree of purity. At least one study using adult CNS neurons to examine neuron-ECM interaction has been published [13], but the author of this review expects to see this technique used more routinely in the future. 

### 2.2. Lesion Models

**Figure 1:**
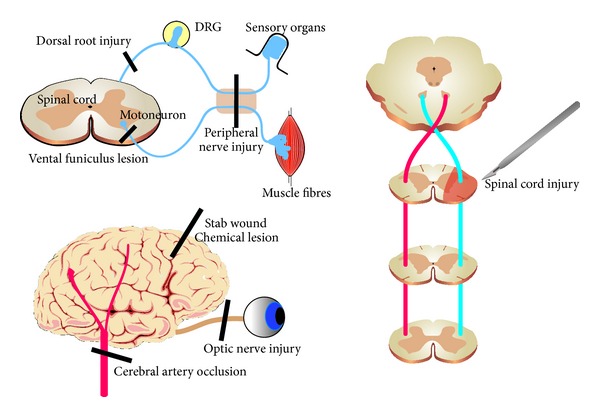
Lesion models discussed in this review.

A number of different lesion models are currently in use. The lesion models mentioned in the text are summarized in Figure 1.

A peripheral nerve injury (compression, crush, or transection) is an injury in the PNS, followed by a regenerative response [2]. Commonly used experimental models are injuries to the sciatic, facial, or laryngeal nerve. After a dorsal root injury, regrowing axons are halted at the border of the spinal cord (the dorsal root entry zone), and functional recovery is not seen [14]. A ventral funiculus lesion is a lesion of the white matter in the ventral part of the spinal cord [15]. This lesion creates an injury of the motoneuron axon in a CNS environment. 

Spinal cord injury models can be either partial, such as dorsal column injury, hemisection (depicted), or complete. In addition, compression and weight-drop models are commonly used [16]. Optic nerve injury (cut or compression) is a lesion of a nerve with CNS biology (oligodendrocytes, etc.) [17]. This lesion axotomizes the retinal ganglion cells which covey visual information from the retina in the eyeball to the lateral geniculate nucleus in the thalamus. Stab wounds create a small, well-defined lesion to study the reaction of neighbouring cells in the CNS [18]. Experimental stroke models are created by occlusion of one of the arteries supplying the brain with oxygenated blood [19]. This lesion is complex both in terms of the graded response observed postinjury (a severely injured lesion core, surrounding penumbra, and uninjured brain tissue) and the different secondary complications that follow, such as inflammation and oedema [19]. Finally, injection of neurotoxins is a useful tool in neurotrauma research. A variety of toxins are used for different purposes. For example, kainic acid is commonly used to mimic seizures or glutamate toxicity that occurs after traumatic brain [20], and injection of LPS (lipopolysaccarides) is used to mimic inflammatory conditions [21]. There are also toxins with a high degree of specificity for certain groups of neurons: 6-hydroxydopamine which kills dopaminergic (and noradrenergic) neurons can be injected into the substantia nigra and is thus used as an animal model for Parkinson's disease [22]. 

## 3. Neuronal ECM Receptors

### 3.1. Integrins

**Figure 2:**
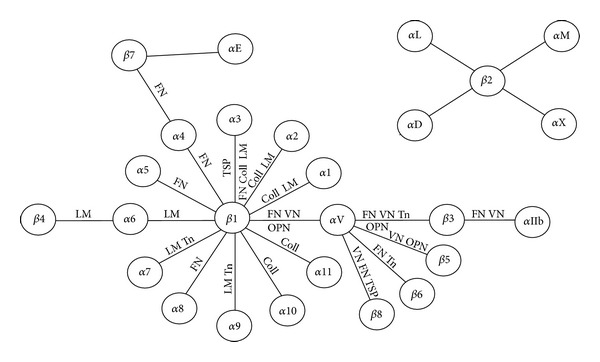
Integrins and ligands discussed in this review. LM: Laminin, Tn: Tenascin, OPN: Osteopontin, FN: Fibronectin, VN: Vitronectin, and TSP: Thrombospondin.

Integrins are expressed on all cell types and phylogenetically conserved. Plants, fungi, and prokaryotes do not express integrin homologues [23]. In mammals, 18 *α* and 8 *β* genes have been identified, and to date 24 different receptors have been identified, see Figure 2. The phenotypes of knockout animals range from very severe to apparently normal, and ten published knockouts are lethal, namely, *α*3, *α*4, *α*5, *α*6, *α*8, *α*9, *α*V, *β*1, *β*4, and *β*8 [24]. The phenotypes of knockout animals have been thoroughly reviewed elsewhere [24–26] and will not be further addressed in this review. Integrins bind a variety of proteins, such as ECM molecules, cell-surface receptors, or blood proteins. Whereas some *α*-*β* combinations display a high degree of specificity in their ligand, others are much more promiscuous. Integrins containing the *β*1 or *α*V subunits bind ECM proteins such as collagen, laminin, vitronectin, osteopontin, and fibronectin, whereas *β*2 integrins, which are found on immune cells, bind cell surface receptors such as ICAMs (intercellular adhesion molecules). Integrins containing *α*4, *α*5, *α*8, *α*IIb, and *α*V subunit bind proteins and peptides carrying the RGD (Arg-Gly-Asp) sequence such as fibronectin and vitronectin [27]. Integrins containing *α*1, *α*2, *α*3, *α*6, and *α*7 have long been known to bind members of the ECM proteins known as laminins. This picture is, however, constantly changing. For example, *α*9*β*1 has been shown to bind laminin [28].

Historically, the ability of integrins to mediate adhesion and cell spreading led to the idea that their function was to serve as a link between the ECM and the cytoskeleton via linker proteins such as talin, paxillin, and *α*-actin, reviewed by [29]. Integrins also play a role in initiating intracellular signalling. Integrins lack catalytic activity, but upon ligand binding, they initiate intracellular signalling cascades by activating kinases such as integrin linked kinase (ILK), focal adhesion kinase (FAK), PI3-kinase, and protein kinase C (reviewed by [23, 29, 30]).

### 3.2. Integrins in the Nervous System

Although expression of integrins has been detected in the developing nervous system [31], information on the expression of these molecules in the adult brain was largely lacking until a very thorough mapping study was performed by Pinkstaff and colleagues [32]. They used *in situ *hybridization to examine the expression of 14 different integrin mRNAs in the adult rat brain and brainstem. Notably, they were unable to detect any expression of integrins *α*2, *β*2, and *β*3 and very restricted expression of *β*4 and *α*4. In contrast, they described a widespread expression of integrins *α*1, *α*3, *α*6, *α*7, *α*V, and *β*1. Motoneurons in the facial nucleus and in the sciatic motor pool express integrins *α*3 and *α*6 and particularly high amounts of *α*7 and *β*1 [32–34]. Dorsal root ganglion (DRG) neurons have been found to express a number of integrins: *α*1, *α*3, *α*4, *α*5, *α*6, *α*7, and *β*1 [34–39]. Readers interested in thorough descriptions of integrin-mediated signalling, and surface to cytoskeletal interactions in neural regeneration are encouraged to examine high quality in-depth reviews of these matters such as [40, 41].

### 3.3. Other ECM Receptors

In addition to integrins, a number of neuronal ECM receptors with functions in neurite outgrowth exist. Dystroglycan binds laminin [42], CD44 binds osteopontin [43] and certain collagen isoforms, and CD47 interacts with thrombospondin. 

## 4. ECM Molecules and Neuronal Regeneration

### 4.1. Laminin

**Figure 3:**
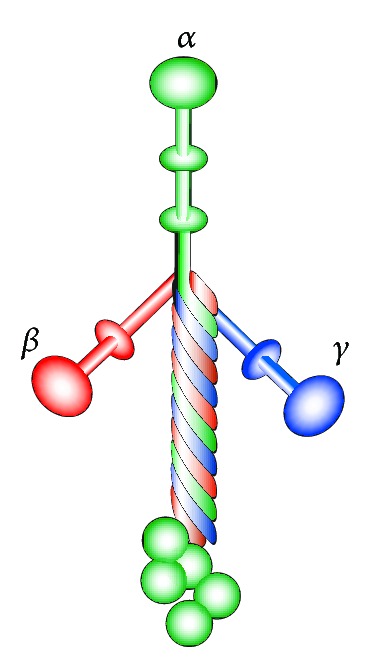
The molecular anatomy of laminins.

Laminins are heterotrimeric proteins composed of one *α*, one *β*, and one *γ* chain. Currently 5 *α*, 3 *β*, and 3 *γ*, chains are known, and 16 *αβγ* combinations have been described [44]. Laminins range in size from approximately 400 to 900 kDa. All laminins have a coiled-coil structure where all three subchains intertwine and a series of five globular domains in the *α* chain C-terminal, see Figure 3. Over the years, the laminin nomenclature has changed several times, with the latest (and probably most convenient) being published in 2005 [44].  Table 1 lists laminin isoforms and chain composition. In detail descriptions of laminin subchains and specific domains can be found in [44, 45].

Laminin immunoreactivity has been detected in areas of axonal growth in the developing CNS and PNS [46, 47]. It is also seen in those CNS areas where adult regeneration is observed, such as the olfactory system [48] in the ventral funiculus after injury, where lesioned axons regenerate over spinal cord scar tissue [49]. Given that this family of proteins has received extensive attention, various isoforms will be addressed individually.

#### 4.1.1. Laminin-111

In 1979, Timpl and coworkers isolated laminin-111 from mouse Engelbreth-Holm-Swarm sarcoma cells [50]; hence the previous name is EHS-laminin. The fact that this was the first isoform to be described and the ease with which it can be purified has led to being regarded as the “prototype” laminin and used extensively. Although available preparations of this laminin isoform are generally of good quality, it can be in complex with nidogen, which may influence physiological properties [51]. 

Expression of laminin *α*1 (possibly forming laminin-111, *α*1*β*1*γ*1) has been detected in trigeminal nerve bundles [52], indicating that Schwann cells in some locations express this subunit. Also, in peripheral nerves of laminin *α*2 deficient mice, laminin *α*1 can be detected [53, 54], but the functional effects of this compensatory upregulation are not yet clear. Expression of this subunit has also been described in Pacinian corpuscles in human skin [55], suggesting that LM-111 could serve as an instructive role in axonal guidance.

#### 4.1.2. Laminin-211

Expression of laminins *α*2, *β*1, and *γ*1 has been detected in both developing and adult peripheral nerves of rodents or human origin [56–58]. These subunits are also upregulated in the proximal stump of injured rat sciatic nerve [58], possibly as part of a regenerative response in providing a substrate for axonal outgrowth. Loss of laminin *α*2 leads to peripheral neuropathy as seen in several animal models such as the dy/dy mice, reviewed by [59], and in the human condition known as merosin-deficient congenital muscular dystrophy (MDCMD) [60]. In the peripheral nerve, the lack of laminin *α*2 is manifested as reduced myelination, discontinuous basal lamina, atypical Schwann cell ensheathment, and abnormal impulse propagation, reviewed in [61]. In addition, LM-211 subunits are also expressed in Meissner's corpuscles in human skin [55]. Several lines of evidence suggest that laminin-211 supports neurite growth under some conditions: cultured embryonic spinal motoneurons from rat extend neurites on LM-211 to a greater extent than on LM-811 or collagen [62], adult DRGs extend neurites on laminin-2 in the presence of nerve growth factor (NGF) [55], Schwann cells from *α*2-deficient mice are less supportive for embryonic DRG growth [61], and addition of *α*2 blocking antibodies reduces growth of DRGs on nerve sections [63]. However, studies of adult regeneration *in vivo*, using any of the laminin *α*2 deficient mice or rat strains, have thus far not been performed. Laminin-211 (merosin) has been available commercially (and used extensively) as a preparation from human placenta, but these preparations have been of suboptimal quality regarding purity and molecular integrity [64]. Protocols for production of recombinant LM-211 have since been developed [65].

#### 4.1.3. Laminin-411

Similar to LM-211, laminin-411 subunits have been detected in peripheral nerves [52, 56–58] and upregulated after injury [58]. LM-411 was purified from a human glioblastoma cell line in 2001 [66], and a recombinant version was created in 2006 [67]. Neonatal trigeminal sensory neurons extend neurites when cultured on LM-411 and LM-111, but not on LM-211, in contrast to the results of spinal motoneurons [62]. Adult DRG neurons grow neurites only slightly better on LM-411 compared to LM-211 [55]. These results indicate possible cell-type specific preference of different neural types for outgrowth. The LM-alpha4 chain is subject to chondroitin sulphate modification [68]. However, according to unpublished results, we did not see any difference in neurite growth when neurons were grown on LM-411 that had been pretreated with chondroitinase. At current, the *in vivo* situation is not clear since studies using laminin *α*4 deficient animals did not show hampered regeneration [62]. However, deletion of the laminin *γ*1 subunit in the peripheral nerve (thus ablating all functional laminin trimers) causes a profound reduction of the regeneration of spinal motoneurons after sciatic nerve crush [69].

#### 4.1.4. Laminin-511

In addition to laminins-211 and -411, expression of laminin *α*5 (thus possibly forming laminin-10, *α*5*β*1*γ*1) has also been described in peripheral nerves [53, 70], but much less abundant. The full function of this isoform in peripheral nerves is not known, but it has been suggested that it may participate in the positioning of sodium channels in the nodes of Ranvier [53]. Similar to *α*2 and *α*4, the *α*5 subunit was also detected in Meissner's corpuscles in human skin [55], and a preparation containing LM-511 enhanced sensory recovery in grafted skin [71]. The function of laminin-511 was initially examined *in vitro *using preparations from human placenta [72], but in order to improve quality recombinant human LM-511 was produced by Doi and colleagues [73]. Of four laminins tested (LM-111, -211, -411, and -511), LM-511 induced the most extensive outgrowth from adult DRG neurons *in vitro* [55], mediated via integrin *α*6*β*1. Spinal motoneurons also grow well on LM-511, but less extensive than those on LM-211 [62]. Recent studies suggest that the neurite growth-promoting properties of laminin-511 are located to the L4a domain [74]. In the CNS, laminin is quite absent from the neuropil, with one exception, the hippocampal formation. The laminin matrix here has a neuroprotective function (since destruction of the laminin matrix is essential for excitotoxicity) [75, 76]. By immunohistochemistry, work from the same lab suggested that laminin 511 is likely the major isoform in the hippocampus [77]. This finding was subsequently expanded on by Fusaoka-Nishioka and colleagues [78], who cultured embryonic hippocampal neurons on laminins-111, -211, -411, and -511 and found 511 to have a growth-promoting capacity greater than the other isoforms. 

#### 4.1.5. Laminins-121 and -221

Sasaki and coworkers [79] recently produced recombinant laminins-121 and -221. In the same study, both isoforms were shown to stimulate growth from adult mouse DRG neurons, and growth on LM-121 was more extensive than EHS-laminin, recombinant laminins-111, and -211. Future studies will likely answer if this isoform also supports growth from other neuronal types.

#### 4.1.6. Exogenous Administration of Laminins after CNS Lesions

So far, exogenous administration of laminins into lesion sites in the CNS has not been able to improve functional regeneration. However, a recent report by Menezes and coworkers showed that a polymerized form of laminin (produced by treating commercially available laminin preparations with a low-pH buffer) supports axonal regeneration and functional recovery after spinal cord injury in rats [80]. In addition, functional recovery has also been seen in spinal cord injury after injection of self-assembling nanofibres that present the laminin-derived IKVAV epitope at high density [81]. Injection of another laminin-derived peptide (KDI) protects dopaminergic neurons from 6-hydroxydopamine-induced injury [82] and improves regeneration and functional recovery after spinal cord injury in rats [83]. 

### 4.2. Fibronectin

Fibronectin (FN) is a large glycosylated protein, composed as a dimer and exists in both a soluble form (e.g., in plasma) and as an ECM constituent in the developing nervous system [47]. Although adult levels are relatively low in the PNS, it undergoes a profound upregulation after injury [84]. FN was first found to support growth from embryonic retinal ganglion cells by Akers and coworkers, [85] and has since then been shown to increase outgrowth from several types of neurons such as embryonic dorsal root ganglion neurons, sympathetic ganglions, and spinal cord neurons [37, 86, 87]. The glycosylation of FN has been shown to affect its ability to support neurite growth [88]. Compared to laminin, FN was a very weak promoter of growth for adult DRG neurons in culture [55], and it should be noted though that after peripheral nerve injury, the FN gene undergoes a dramatic change in splicing and several different isoforms are produced, some with a higher potential to support growth [39, 89], indicating that caution should be exercised when extrapolating results from cell-culture studies. Using a culture system where adult DRG neurons are grown on slices of CNS white matter, Tom and colleagues [90] found that blocking antibodies raised towards FN decreased neurite growth indicating that this molecule could be of importance for regeneration in the CNS. This hypothesis has been further supported by a recent report showing that cultured adult CNS neurons (from cortex and hippocampus) grew better on FN than laminins-111 or -112 [13]; however, laminin-511 (the most potent stimulator of CNS neurite growth [78]) was not included in this comparison. In addition, a single injection of FN (at the lesion site) after dorsal crush injury led to an increase in sprouting of descending serotonergic fibres and alleviated injury-induced allodynia [91]. Finally, fibronectin has some other interesting qualities. Using a culture system where FN is attached to conducting polymers, Svennersten and colleagues [92] showed that when the molecule is a reduced state (but not in oxidized), RGD sites are exposed and effect cell attachment and proliferation. On a different, but related, note, stretching of FN has also been shown to cause exposure of cryptic binding sites [93]. How these processes might be exploited for neuronal outgrowth remains to be explored.

### 4.3. Osteopontin

Osteopontin (OPN) is a glycosylated phosphoprotein of 44 kDa. It also goes by the names secreted phosphoprotein 1 (SPP1), bone sialoprotein 1 (BSP-1), and early T-lymphocyte activation-1 (ETA-1). It was originally identified as a component of the ECM of bone tissue (hence its name derived from the Latin words “osteo” bone and “pons” bridge). The OPN protein harbours the RGD motif [94]. 

Upregulation of OPN has been detected after cerebral ischemia [95], focal brain injury [96, 97], spinal cord injury [98], and optic nerve crush [99]. *In vitro* studies of OPN's effect on DRG neurite growth are somewhat inconclusive with either no reported effect [100] or inhibitory effect [101]. Concerning the CNS, there is evidence suggesting a supportive role of OPN: cultured retinal ganglion cells grow well on OPN [43, 102], and a recent report by the author of this review shows that cultured hippocampal neurons also grow well on this substrate [97]. Further, Hashimoto and colleagues reported impaired regenerative growth of corticospinal fibres after spinal cord injury in OPN −/− mice compared to wild type [103]. The authors also demonstrated impaired functional recovery in these mice, but the underlying mechanism is not completely understood. Finally, OPN also shows promise as a neuroprotective agent, as administration of this molecule protects neuronal cultures from ischemic injury *in vitro* [104], decreases infarct size after experimental stroke in mice [104], and promotes recovery after intracerebral haemorrhage [105].

### 4.4. Vitronectin

In terms of neurite growth, vitronectin (VN) has mainly been studied in regard to the visual system. Retinal ganglion cells extend neurites when cultures on VN [106, 107] and VN are upregulated after optic nerve crush in adult animals [108]. Also VN supports neurite growth from DRG neurons, on par with laminin and to a higher degree than fibronectin [109]. Further, postnatal cerebellar granule cells extend neurites in response to VN, via interaction with the RGD site [110]. In contrast, a recent study by Previtali and colleagues [111] showed that VN was upregulated in nerves from patients with defective regeneration due to peripheral neuropathy (whereas the levels of laminin and collagen IV were similar), and when compared for outgrowth-stimulating properties, VN was a poor substrate compared to collagen and fibronectin.

### 4.5. Thrombospondins

Thrombospondin-1 (TSP-1) supports growth from several types of neurons in culture: retinal ganglion cells [106], superior cervical ganglion neurons [106, 112, 113]. It is upregulated in peripheral nerve after injury and appears to support neuronal growth [114]. In addition, TSP-1 is upregulated at the injury site after spinal cord injury [115] and experimental stroke in mice [116] and rats [117]. TSP knockout mice display deceased neuronal sprouting and impaired functional recovery after stroke [116]. Another TSP family member, thrombospondin-4, has also been shown to support growth from several types of neurons, using a coculture with cells over expressing this molecule [118]. Later studies by Dunkle and coworkers showed that TSP-4 is expressed in the developing retina, and although it does not by itself support growth of retinal ganglion cells, it does potentiate the outgrowth-promoting properties of laminin [119]. TSP-4 is also produced in the dorsal horn of the spinal cord (likely by reactive astrocytes), in neuropathic pain models, and infusion of TSP-4 caused allodynia in rats [120], possibly by stimulating aberrant sprouting or synaptic plasticity.

### 4.6. Tenascin-C

Tenascins (Tn) are a family consisting of five members (Tenascins-C, -R, -X, -Y, and -W), where tenascin-C is the most extensively studied with regard to neuronal outgrowth and regeneration [121]. *In vitro* studies using embryonic sensory and motor neurons [122] and cerebellar granule neurons [123] indicate that TnC could be beneficial for outgrowth. *In vivo*, tenascin-C is upregulated after ventral funiculus lesion [124], and studies using knockout mice, forced overexpression, and protein infusion indicate a beneficial effect on regeneration after spinal cord injury [125]. In contrast Andrews and coworkers [126] did not observe extensive outgrowth from cultured DRG neurons on TnC, but having observed an increase in TnC in CNS scar tissue they overexpressed integrin *α*9*β*1 in DRG neurons and observed regeneration of fibres after dorsal root injury.

## 5. Conclusion 

This review summarizes the supportive functions of various ECM molecules from the perspective of neurite outgrowth. Needless to say, ECM molecules also influence other aspects of nervous system regeneration such as synapse formation [127, 128] and migration of neural stem cells [129] or cells transplanted to the CNS [130] that are not addressed in this review. 

Further, in addition to the aforementioned nanofibres [81], novel developments in material design, using either incorporated ECM motives [131] or coupled intact molecules such as laminin [132], will hopefully be able to provide the research community with new tools to develop strategies to improve neural regeneration.

Further, the complex interplay between supportive and inhibitory molecules is not yet fully understood. Although laminin can block the inhibitory effect of myelin-associated glycoprotein (MAG) [133], it can also switch the influence of netrin from attractive to repulsive [134] and mediate the inhibitory effect of ephrin5 [135]. These examples show that the practise of segregating molecules into either “inhibitory” or “stimulating” may be to oversimplify the situation. Perhaps improved knowledge about this will increase our chances of designing successful therapies for CNS injuries. 

Finally, when considering regeneration *in vivo*, it is important to remember that within both the CNS and the PNS there is a great heterogeneity concerning regenerative ability. There are several examples of CNS neurons that do regenerate given the right circumstances [136–138], and some PNS neurons seem to possess less of a regenerative capacity than others [139]. From this perspective, it seems unlikely that one ECM molecule will be able to support growth from all types of neurons and that increased knowledge on different subgroups of neurons and their preferred substrate for growth is highly desirable.

## Figures and Tables

**Table 1:** Laminin chain composition and nomenclature.

Laminin	Chain composition	New nomenclature [44]
Laminin-1	*α*1*β*1*γ*1	111
Laminin-2	*α*2*β*1*γ*1	211
Laminin-3	*α*1*β*2*γ*1	121
Laminin-4	*α*2*β*2*γ*1	221
Laminin-5 or 5A	*α*3A*β*3*γ*2	332 or 3A32
Laminin-5B	*α*3B*β*3*γ*2	3B32
Laminin-6 or 6A	*α*3A*β*1*γ*1	311 or 3A11
Laminin-7 or 7A	*α*3A*β*2*γ*1	321 or 3A21
Laminin-8	*α*4*β*1*γ*1	411
Laminin-9	*α*4*β*2*γ*1	421
Laminin-10	*α*5*β*1*γ*1	511
Laminin-11	*α*5*β*2*γ*1	521
Laminin-12	*α*2*β*1*γ*3	213
Laminin-14	*α*4*β*2*γ*3	423
—	*α*5*β*2*γ*2	522
Laminin-15	*α*5*β*1*γ*3	523

## Abbreviations

CNS: Central nervous system
DRG: Dorsal root ganglion
ECM: Extracellular matrix
FAK: Focal adhesion kinase
FN: Fibronectin
EHS: Engelbreth-Holm-Swarm sarcoma
ICAM: Intercellular adhesion molecule
ILK: Integrin linked kinase
LM: Laminin
MAG: Myelin-associated glycoprotein
NGF: Nerve growth factor
OPN: Osteopontin
PNS: Peripheral nervous system
Tn: Tenascins
TSP: Thrombospondin
VN: Vitronectin.
